# Epidemiology of thyroid cancer in Asia in 2020 and its projection to 2040

**DOI:** 10.1186/s12889-025-24029-9

**Published:** 2025-09-30

**Authors:** Seyed Ehsan Mousavi, Negin Abiri Jahromi, Kimia Motlagh Asghari, Seyed Aria Nejadghaderi

**Affiliations:** 1 Neurosciences Research Center, Aging Research Institute, Tabriz University of Medical Sciences, Tabriz, Iran; 2 Department of Community Medicine, Social Determinants of Health Research Center, Faculty of Medicine, Tabriz University of Medical Sciences, Tabriz, Iran; 3 Tehran University of Medical Sciences, Tehran, Iran; 4 Immunology Research Center, Tabriz University of Medical Sciences, Tabriz, Iran; 5 HIV/STI Surveillance Research Center, and WHO Collaborating Center for HIV Surveillance, Institute for Futures Studies in Health, Kerman University of Medical Sciences, Kerman, Iran; 6 Knowledge Hub for Migrant and Refugee Health, Institute for Futures Studies in Health, Kerman University of Medical Sciences, Kerman, Iran

**Keywords:** Thyroid neoplasm, GLOBOCAN, Epidemiology, Asia, Incidence, Mortality, Prevalence

## Abstract

**Background:**

Thyroid cancer has incidence continues to rise globally. Understanding the epidemiology of thyroid cancer and the future burden on public health systems is necessary. We aimed to investigate the prevalence, incidence, and mortality of thyroid cancer by age, sex, country in Asia in 2020 and its projection up to 2040.

**Methods:**

The Global Cancer Observatory provided data on thyroid cancer incidence and mortality for the year 2020. By taking into account the sex, age group, and Asia region, the counts, rates, and mortality-to-incidence ratios (MIRs) were calculated. To estimate the projected number of new cancer cases or mortalities between 2025 and 2040 in a particular nation, the corresponding expected population for the years 2025 to 2040 was multiplied by the age-specific incidence or mortality rates calculated for 2020.

**Results:**

In 2020, the 5-year prevalence rate, age-standardized incidence rate (ASIR), and age-standardized mortality rate (ASMR) for thyroid cancer were 24.60, 6.40, and 0.44 per 100,000 population in Asia, respectively. By country, the highest thyroid cancer 5-year prevalence rates and ASIRs were found in the Republic of Korea. The highest thyroid cancer ASMRs among both sexes combined were in the United Arab Emirates and Syrian Arab Republic. The highest incidence and mortality crude rates were in the 50–59 and 70 + age groups, respectively. Women had higher incidence and mortality rates than men. There were significant correlations between human development index and MIR and ASIR (*p* < 0.001). The number of newly diagnosed thyroid cancer cases and mortalities are expected to rise by 26.6% and 77.8% up to 2040 in Asia, respectively.

**Conclusions:**

Asia is experiencing rising rates of thyroid cancer incidence and mortality. It is imperative to prioritize strategies aimed at high-incidence regions, transitioning countries with limited resources, and younger adult populations to alleviate the global burden of thyroid cancer and resolve disparities in cancer management.

**Supplementary Information:**

The online version contains supplementary material available at 10.1186/s12889-025-24029-9.

## Introduction

Thyroid cancer is increasingly globally, almost due to its surging incidence and the overdiagnosis [1]. In recent decades, thyroid cancer has risen to become one of the most rapidly proliferating malignancies worldwide, with Asia mirroring this concerning trajectory [2]. In 2020, thyroid cancer was among top ten cancers in terms of incidence worldwide [3]. Also, there are several types of thyroid cancers (e.g., papillary, follicular, medullary, and anaplastic), each characterized by distinct histological subtypes, thereby introducing unique challenges in terms of diagnosis, treatment, and prognosis [4–6]. Several factors like genetic, environmental exposures, and evolving diagnostic practices play roles in the increase of thyroid cancer incidence [2, 7]. The highest incidence rates are often observed in countries with a high prevalence of iodine deficiency or excess, emphasizing the pivotal role of iodine in thyroid health [8–11].

Based on data from the global burden of disease (GBD) study in 2019, the mortalities were more than 45 thousands globally [12]. The incidence of thyroid cancer differed among regions and countries, with higher rates observed in countries with middle and high socioeconomic development. Additionally, women experienced higher incidence rates of thyroid cancer than men [12]. With its diversity and varying healthcare landscapes, Asia is a unique continent for understanding the regional dynamics influencing thyroid cancer epidemiology [13]. According to GBD 2019, the age-standardized incidence rates (ASIRs) of thyroid cancer in Central, Southeast, and East Asia were 1.69, 3.72, and 2.11 per 100000, respectively [12]. The 5-year survival rate for thyroid cancer in Asian countries in 2022 was 95.3% [14]. Compared to European countries, the survival rate for thyroid cancer in Asian countries was higher. However, it was lower than the survival rate in the United States [14]. Moreover, the impact of increased access to healthcare and improved diagnostic techniques cannot be overlooked as contributors to the observed rise in incidence rates. Thyroid cancer exhibits a notable gender disparity, with women being more frequently affected than men [15]. This discrepancy is particularly evident in the prevalence of papillary thyroid carcinoma, the most common histological subtype, which disproportionately affects women [15].

Previous studies on the epidemiology of thyroid cancer have shed Light on various aspects of this disease, providing valuable insights into its prevalence, mortality rates, and associated factors. One study performed a comprehensive assessment of the global distribution of thyroid cancer incidence and mortality rates in 2020 [2]. The capstone paper of Global Cancer Observatory (GLOBOCAN) provided an update on the global cancer burden, including thyroid cancer, while it was not focused on thyroid cancer [3]. They highlighted the increasing incidence rates in transitioning countries and emphasized the importance of building sustainable infrastructure for cancer prevention and care [3]. The GBD 2019 study investigated the temporal patterns of incidence, mortality, and disability of various cancers in Asia, offering insights into the changing landscape of cancer burden and its associated risk factors [16]. Additionally, another study using the GBD 2019 data reported the burden of this cancer in North Africa and the Middle East [17]. Another study using the GBD 2019 data also reported thyroid cancer burden in Asia in 2019 [18]. However, these studies were not focused on Asia or the data are out of dated. Although previous studies have provided valuable insights, our study brings a unique perspective in several ways. Firstly, we offer a comprehensive analysis of thyroid cancer epidemiology across multiple Asian countries, which includes diverse populations and healthcare settings. Secondly, we have used the GLOBOCAN 2020 database, which provides updated estimates and projections for the thyroid cancer burden in Asia. We have implemented an updated approach that includes age-standardized rates and stratification based on the human development index (HDI). Our goal was to examine the prevalence, incidence, and mortality of thyroid cancer across different age groups, sexes, and countries in Asia during the year 2020. Additionally, we aimed to project these statistics up to the year 2040. This allows for more accurate comparisons and interpretations of thyroid cancer trends within the region.

## Methods

### Data sources

Information regarding thyroid cancer (classified as international classification of disease (ICD)−10: C73) epidemiology was acquired from GLOBOCAN, a publicly accessible database administered by the International Agency for Research on Cancer and the World Health Organization. GLOBOCAN 2020 offers current estimates of cancer epidemiology, covering 36 primary cancer types categorized by sex and age across 185 countries and 30 global regions [19]. In earlier research, comprehensive explanations of both the data sources and the hierarchy of methods used have been provided [3, 20]. Various techniques were employed to estimate the incidence and mortality rates specific to age, sex, and country. These methods included projecting the observed national incidence and mortality rates for the year 2020, applying recent incidence and mortality rates to the 2020 population, using modeling to obtain rates from national mortality data based on mortality-to-incidence ratios (MIRs) derived from cancer registries in the country, determining rates through modeling using MIRs from cancer registries in neighboring countries, calculating age- and sex-specific national incidence rates for all cancers by averaging overall rates from neighboring countries, and finally, deriving rates as an average of those from selected neighboring nations [20]. The sex, age, and country-specific incidence-to-five-year prevalence ratios from Nordic countries between 2006 and 2015 were used to calculate the 5-year prevalence estimates of thyroid cancer.

$${Prevalenc}_{Country}= {Incidence}_{Country}\times \frac{{Prevalenc}_{Nordic}}{{Incidence}_{Nordic}} \times \frac{{HDI}_{Country}}{{HDI}_{Nordic}}$$

Population statistics for 2020 were sourced from the 2019 edition of the United Nations World Population Prospects. HDI data were obtained from the United Nations Development Programme's Human Development Report Office [21]. Data on current healthcare expenditure (CHE) as a percentage of gross domestic product (GDP) for 2019 were extracted from the World Health Organization's Global Health Observatory data repository [22]. This study adhered to the Guidelines for Accurate and Transparent Health Estimates Reporting statement [23] and followed the Strengthening the Reporting of Observational Studies in Epidemiology statement guidelines [24].

### Study variables

Study variables encompassed two key measures of thyroid cancer: incidence and mortality, which were further refined by calculating their respective crude rates and subsequent MIRs. MIRs serve as indicators of healthcare quality, with lower values indicative of superior cancer care encompassing screening, therapy, and overall disease management [25]. Additionally, the study evaluated cumulative risk percentages for both incidence and mortality of thyroid cancer, providing insights into the risk of developing and succumbing to the disease before the age of 75 years. Another variable examined was CHD as a percentage of GDP (CHE/GDP%), a metric used to gauge the allocation of financial resources to the health sector within a nation, underscoring the sector's importance to the broader economy [22]. Furthermore, the HDI was leveraged to offer a comprehensive measure reflecting the socioeconomic status of countries, drawing on key dimensions of human development such as Life expectancy at birth, educational attainment by individuals aged 25 years and older, the projected number of years of education for school-age children and gross national income per capita in purchasing power parity [21].

### Statistical analysis

We presented comprehensive tables and figures illustrating various metrics including the number of new cases and mortalities, 5-year prevalent cases, crude incidence and mortality rates, 5-year prevalence rate, age-standardized incidence (ASIR), and age-standardized mortality rate (ASMR), all standardized per 100000 population. To ensure accurate comparisons across populations with differing age distributions, we employed the direct standardization method using the Segi-Doll World standard population from 1966. Furthermore, we explored the relationship between thyroid cancer incidence and mortality rates, along with the estimated MIR, in relation to the HDI and CHE/GDP% for countries with available data. This analysis was conducted using bivariate correlation tests, with results reported using Pearson's correlation coefficient categorized into strong (> 0.5), moderate (0.5–0.3), and weak (< 0.3) ranges based on its absolute value. Statistical significance was determined by a p-value of less than 0.05 from a two-sided test. Additionally, we projected the number of new cancer cases or mortalities between 2025 and 2040 by extrapolating age-specific incidence or mortality rates calculated for 2020 and applying them to the corresponding expected population for the years 2025 to 2040. Population estimates were sourced from the United Nations, World Population Prospects, 2019 revision (https://population.un.org/wpp/). Data analysis was conducted using R statistical software, version 4.3.2 [26].

## Results

### Prevalence, incidence, and mortality at the global, regional, and national levels

Globally, the 5-year prevalent cases of thyroid cancer in 2020 were 1984927, corresponding to a 5-year prevalence rate of 25.50. There were a total of 586202 new cases of thyroid cancer in the world, presenting a crude rate of 7.50 per 100000 population, an ASIR of 6.60, and an all-age cumulative risk of 0.87% among both sexes. Moreover, thyroid cancer led to 43646 mortalities with a crude rate of 0.56, an ASMR of 0.43, and a cumulative risk of 0.14%. Continents with the highest ASIRs for thyroid cancer were Northern America (12.40) and Oceania (9.70), whereas Africa (2.00) was the continent with the lowest ASIR. In contrast, Northern America (0.30) and Europe (0.33) had the lowest ASMRs, while Africa (0.62) accounted for the highest ASMR among continents (Table 1).

**Table 1:** Thyroid cancer five-year prevalence, incidence, and mortality metrics, as well as mortality-to-incidence ratio in 2020 for different geographic location in both sexes, males, and females

Location	Prevalence		Incidence				Mortality				MIR
	5-year prevalent cases	5-year prevalence rate	Number	Crude rate	ASR	Cumulative risk (%)	Number	Crude rate	ASR	Cumulative risk (%)	
Both sexes											
World	1984927	25.5	586202	7.5	6.6	0.87	43646	0.56	0.43	0.14	0.07
Asia	1139172	24.6	349897	7.5	6.4	0.83	25668	0.55	0.44	0.15	0.07
Eastern Asia	868943	51.8	260692	15.5	11.5	1.31	12023	0.72	0.4	0.14	0.05
South-Central Asia	90892	4.5	32548	1.6	1.6	0.26	6983	0.35	0.38	0.11	0.22
South-Eastern Asia	97851	14.6	32629	4.9	4.4	0.74	4578	0.68	0.63	0.25	0.14
Western Asia	81486	29.3	24028	8.6	8.6	1.1	2084	0.75	0.83	0.27	0.09
Continents											
Africa	47595	3.6	18457	1.4	2	0.35	4443	0.33	0.62	0.22	0.24
Europe	325708	43.5	87162	11.6	8.3	0.99	6399	0.85	0.33	0.11	0.07
Latin America and the Caribbean	209541	32	63368	9.7	8.6	1.07	4406	0.67	0.53	0.17	0.07
Northern America	243888	66.1	62256	16.9	12.4	1.58	2420	0.66	0.3	0.1	0.04
Oceania	19023	44.6	5062	11.9	9.7	1.29	310	0.73	0.45	0.13	0.06
Males											
World	438719	11.2	137287	3.5	3.1	0.46	15906	0.4	0.35	0.11	0.11
Asia	259942	11	84355	3.6	3.1	0.44	9737	0.41	0.36	0.11	0.11
Eastern Asia	196578	23	61997	7.2	5.4	0.66	4210	0.49	0.3	0.1	0.07
South-Central Asia	22976	2.2	8851	0.85	0.89	0.16	3361	0.32	0.37	0.11	0.38
South-Eastern Asia	23356	7	8203	2.5	2.4	0.41	1482	0.44	0.47	0.18	0.18
Western Asia	17032	11.7	5304	3.6	3.8	0.57	684	0.47	0.59	0.19	0.13
Continents											
Africa	9039	1.3	3839	0.57	0.94	0.18	1106	0.17	0.34	0.12	0.3
Europe	67958	18.8	19345	5.3	3.7	0.49	2359	0.65	0.31	0.09	0.12
Latin America and the Caribbean	36601	11.4	11866	3.7	3.4	0.47	1445	0.45	0.39	0.12	0.12
Northern America	60417	33.1	16560	9.1	6.3	0.94	1150	0.63	0.32	0.1	0.07
Oceania	4762	22.3	1322	6.2	4.9	0.78	109	0.51	0.32	0.1	0.08
Females											
World	1546208	40	448915	11.6	10.1	1.25	27740	0.72	0.5	0.17	0.06
Asia	879230	38.8	265542	11.7	9.9	1.21	15931	0.7	0.52	0.18	0.06
Eastern Asia	672365	81.7	198695	24.2	17.8	1.94	7813	0.95	0.48	0.17	0.04
South-Central Asia	67916	7	23697	2.4	2.4	0.35	3622	0.37	0.38	0.11	0.15
South-Eastern Asia	74495	22.3	24426	7.3	6.4	1	3096	0.93	0.76	0.3	0.13
Western Asia	64454	48.6	18724	14.1	14	1.66	1400	1.1	1.1	0.33	0.08
Continents											
Africa	38556	5.7	14618	2.2	3.1	0.49	3337	0.5	0.85	0.29	0.23
Europe	257750	66.6	67817	17.5	12.8	1.44	4040	1	0.35	0.12	0.06
Latin America and the Caribbean	172940	52	51502	15.5	13.4	1.61	2961	0.89	0.63	0.21	0.06
Northern America	183471	98.5	45696	24.5	18.4	2.19	1270	0.68	0.28	0.1	0.03
Oceania	14261	66.9	3740	17.5	14.5	1.79	201	0.94	0.57	0.15	0.05

*Abbreviations*: *ASR* Age-standardized rate, *MIR* Mortality-to-incidence ratio. Rates are presented per 100,000 population

In Asia, the 5-year prevalent cases of thyroid cancer reported for 2020 were 1139172, ranking it the highest among all continents. The 5-year prevalence rate was reported 24.60. There were a total of 349897 new cases of thyroid cancer in Asia in 2020, presenting a crude rate of 7.50 per 100000 population, an ASIR of 6.40, and an all-age cumulative risk of 0.83% among both sexes. In 2020, thyroid cancer caused 25668 mortalities in Asia, with a crude rate of 0.55, an ASMR of 0.44, and a cumulative risk of 0.15%. In 2020, among the four Asian regions for both sexes, Eastern Asia had the highest 5-year prevalence rate (51.80), while South-Central Asia had the lowest one (4.50). Similarly, Eastern Asia and South-Central Asia with ASIRs of 11.50 and 1.60 had the highest and lowest ASIRs, respectively. In terms of ASMRs, Western Asia had the highest value (0.83), while South-Central Asia had the lowest one (0.38) (Table 1).

**Fig. 1:**
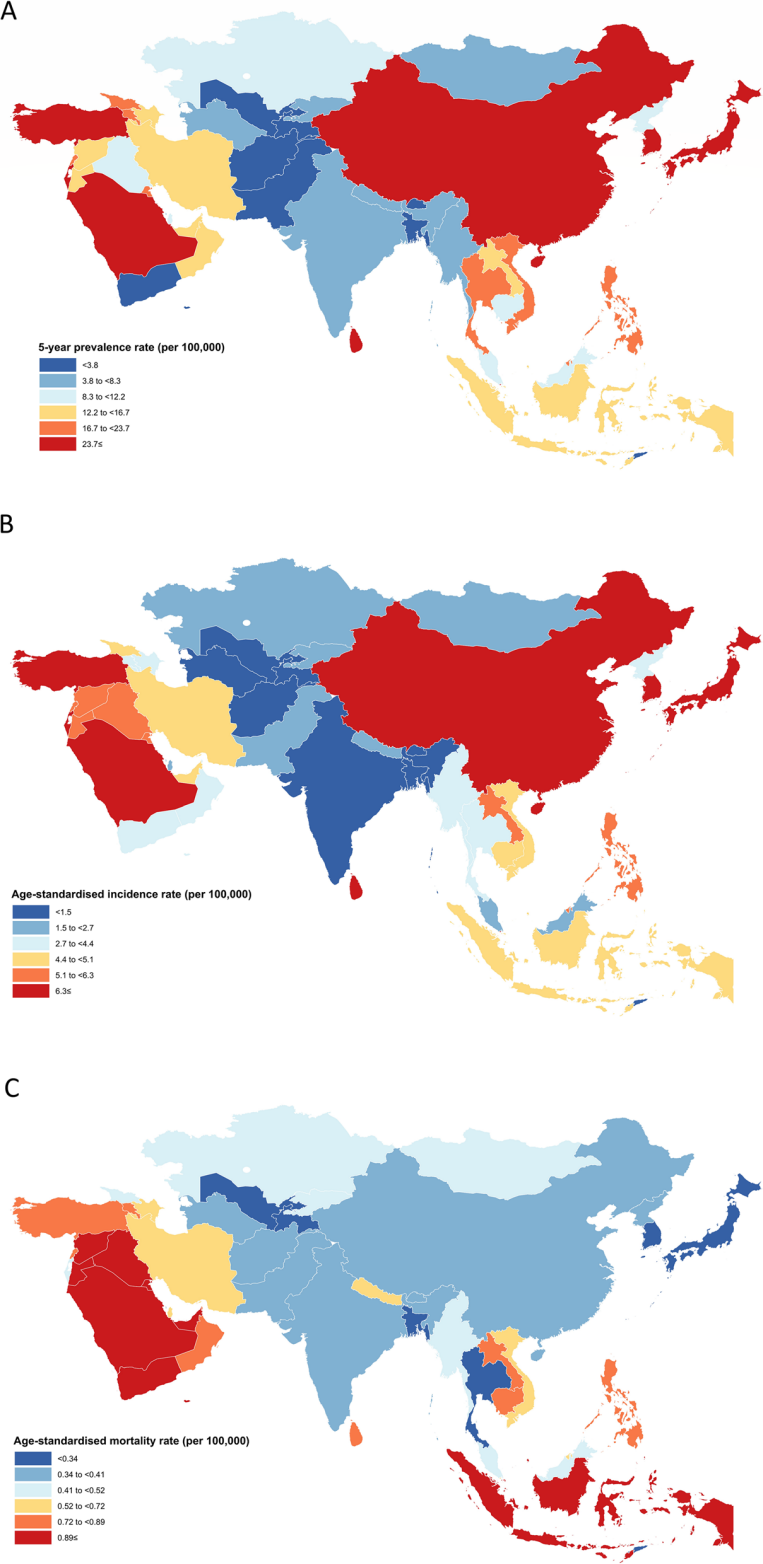
Distribution of (**A**) five-year prevalence rate, (**B**) age-standardized incidence and (**C**) age-standardized mortality rates per 100,000 of thyroid cancer among both sexes in Asia in 2020

Among all the Asian countries, the highest thyroid cancer 5-year prevalence rates were found for the Republic of Korea (120.10), Israel (62.00), and Turkey (56.80). Countries with the lowest 5-year prevalence among both sexes were Tajikistan (0.81) and Afghanistan (1.40) (Fig. 1A). Correspondingly, the Republic of Korea (26.60), Israel (14.30), and Turkey (14.30) had the highest ASIRs per 100000, while Tajikistan (0.40) had the lowest, followed by Bangladesh (0.95) and Afghanistan (1.10) (Fig. 1B). Countries with the highest thyroid cancer ASMRs among both sexes combined were the United Arab Emirates (1.30) and Syrian Arab Republic (1.30), while the rates were lowest in Tajikistan (0.15), Timor-Leste (0.20), Uzbekistan (0.22), and Bangladesh (0.26) (Fig. 1C and Supplementary File 1).

### MIRs of thyroid cancer in Asia

In 2020, the MIR of thyroid cancer in Asia among both sexes was 0.07, which was similar to the world. South-Central Asia (0.22) demonstrated the highest MIR among both sexes, followed by South-Eastern Asia (0.14), Western Asia (0.09), and Eastern Asia (0.05). Among Asian countries, Tajikistan ranked top (0.29), while the Republic of Korea reported the lowest MIR (0.02), followed by China (0.04), and Israel (0.05) (Table 1 and Supplementary File 1).

The MIR of thyroid cancer was 0.11 in men and 0.06 in women worldwide and in Asia. South-Central Asia had the highest, and Eastern Asia had the lowest MIR in both men and women. Among women, Tajikistan (0.28) had the highest, and the Republic of Korea (0.02) had the lowest MIRs. Among men, Bhutan (0.67) had the highest and the Republic of Korea (0.04) had the lowest MIRs (Table 1 and Supplementary File 1).

### Age pattern of thyroid cancer in Asia

In Asia, the 50 to 59 age group had the highest thyroid cancer incident cases (85267) and crude rates of incidence (16.00) among all age groups in 2020 among both sexes, while the 0 to 9 age group had the lowest incident cases (256) and crude rates (0.04). The highest number (12709), crude rate (5.10), and cumulative risk (0.12%) of mortality were observed in the 70 + age group. A similar pattern was seen in the mortality rates for both males and females (Table 2).

**Table 2:** Thyroid cancer incidence, mortality, and mortality-to-incidence ratio metrics in Asia in 2020 for different age groups among both sexes, males, and females

Age group	Incidence			Mortality			MIR
	Number	Crude rate	Cumulative risk (%)	Number	Crude rate	Cumulative risk (%)	
Both sexes							
0 to 9	256	0.04	0	53	0.01	0	0.25
10 to 19	5421	0.75	0.01	245	0.03	0	0.04
20 to 29	29332	4.1	0.04	347	0.05	0	0.01
30 to 39	61499	8.5	0.09	806	0.11	0	0.01
40 to 49	80581	13	0.13	1623	0.26	0	0.02
50 to 59	85267	16	0.16	3607	0.68	0.01	0.04
60 to 69	54771	15.3	0.15	6278	1.7	0.02	0.11
70 +	32761	13.3	0.25	12709	5.1	0.12	0.38
Males							
0 to 9	84	0.02	0	19	0.01	0	0.5
10 to 19	983	0.26	0	77	0.02	0	0.08
20 to 29	6597	1.8	0.02	106	0.03	0	0.02
30 to 39	14247	3.8	0.04	248	0.07	0	0.02
40 to 49	18345	5.8	0.06	569	0.18	0	0.03
50 to 59	20358	7.6	0.08	1632	0.61	0.01	0.08
60 to 69	14691	8.3	0.08	2780	1.6	0.02	0.19
70 +	9050	8.2	0.16	4306	3.9	0.09	0.48
Females							
0 to 9	181	0.05	0	34	0.01	0	0.2
10 to 19	4439	1.3	0.01	168	0.05	0	0.04
20 to 29	22735	6.6	0.07	241	0.07	0	0.01
30 to 39	47252	13.5	0.14	558	0.16	0	0.01
40 to 49	62236	20.6	0.21	1054	0.35	0	0.02
50 to 59	64909	24.6	0.25	1975	0.75	0.01	0.03
60 to 69	40080	22.1	0.22	3498	1.9	0.02	0.09
70 +	23711	17.3	0.32	8403	6.1	14	0.35

*Abbreviations*: *MIR* Mortality-to-incidence ratio

**Fig. 2:**
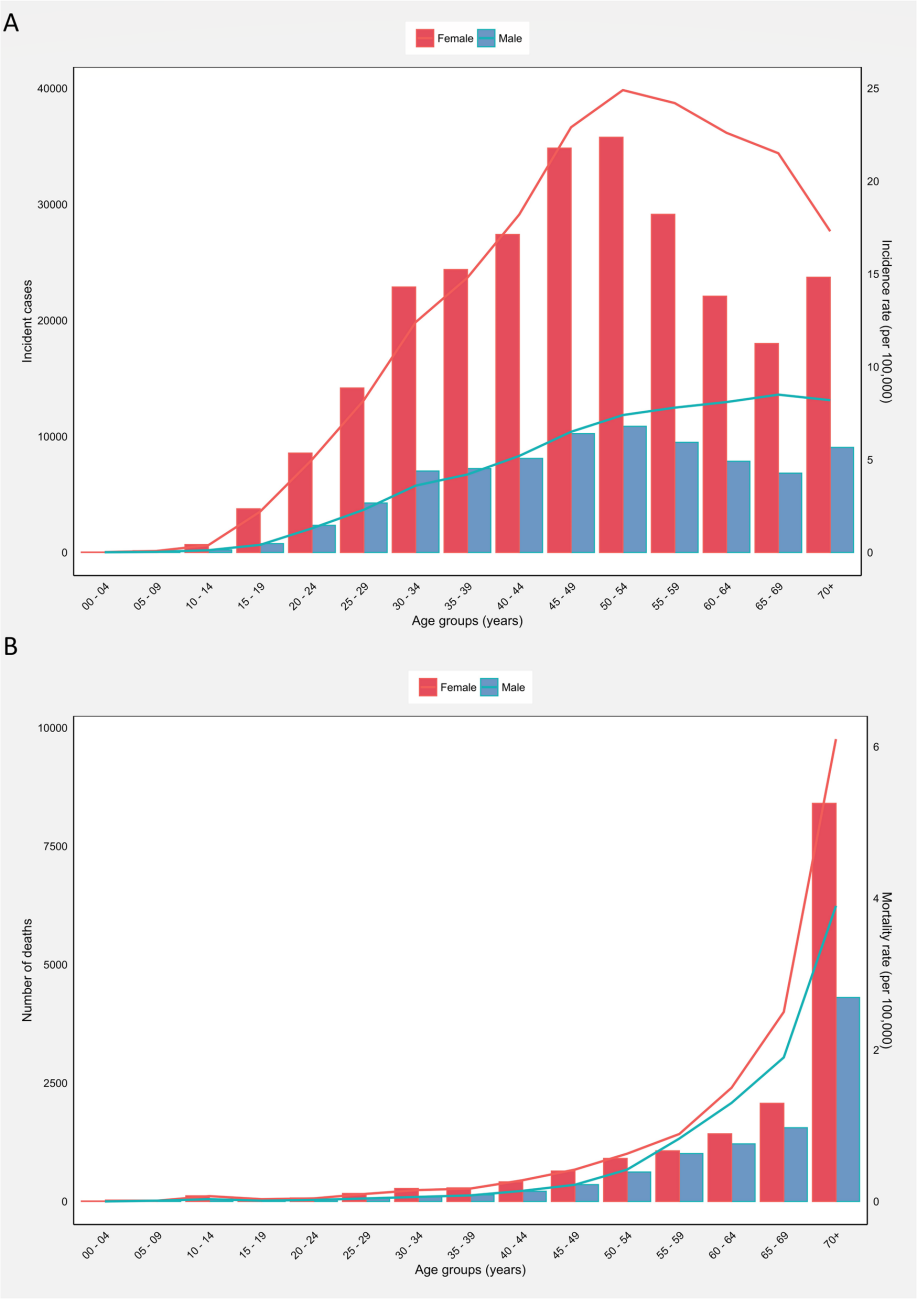
**A** Incident numbers and incidence rate, and (**B**) mortality numbers and mortality rate of thyroid cancer among males and females in each age group in Asia in 2020

Accordingly, the incidence rates of thyroid cancer among males increased with age up to 65–69 age groups and it was increased up to 50–54 age groups for females, before decreasing then after (Fig. 2A). On the other hand, the mortality rates had been continuously increasing and reached their peak in the age group above 70 years (Fig. 2B). Incidence and mortality numbers and rates were generally higher in women than men (Fig. 2A and B).

### Sex pattern of thyroid cancer in Asia

In 2020, the 5-year prevalence rates of thyroid cancer in Asia in women and men were 38.80 and 11.00, respectively. The number of new cases of thyroid cancer was 265542 in women and 84355 in men, corresponding to ASIRs of 9.90 per 100000 for women and 3.10 per 100000 for men. ASMRs for this cancer were 0.52 per 100000 women and 0.36 per 100000 men In Asia (Table 1).

**Fig. 3:**
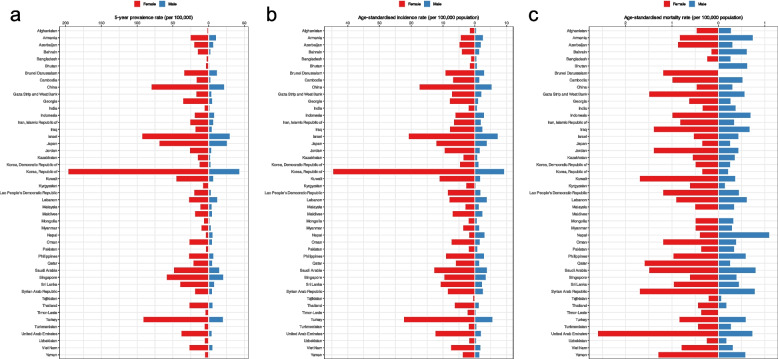
**A** Five-year prevalence rate, (**B**) age-standardized incidence and (**C**) age-standardized mortality rates per 100,000 of thyroid cancer in Asia in 2020, by country and sex

The 5-year prevalence rates and ASIRs of thyroid cancer in women (196.80 and 45.00) and men (43.60 and 9.40) were highest in the Republic of Korea among all Asian countries (Figure S1 and Figure S2). While, the lowest 5-year prevalence rates and ASIRs in women (1.30 and 0.60) and men (0.29 and 0.20) were in Tajikistan (Fig. 3A, B, Figure S3 and Figure S4). The United Arab Emirates had the highest mortality rate in women, with an ASMR of 2.60 per 100000, and Nepal had the highest mortality rate in men, with an ASMR of 1.10 per 100000 population. Bahrain (0.16) and Tajikistan (0.07) had the lowest ASMRs among women and men, respectively (Fig. 3C, Figure S5, Figure S6, and Supplementary File 1).

### Correlations between incidence, mortality, MIR, HDI, and CHE/GDP

**Fig. 4:**
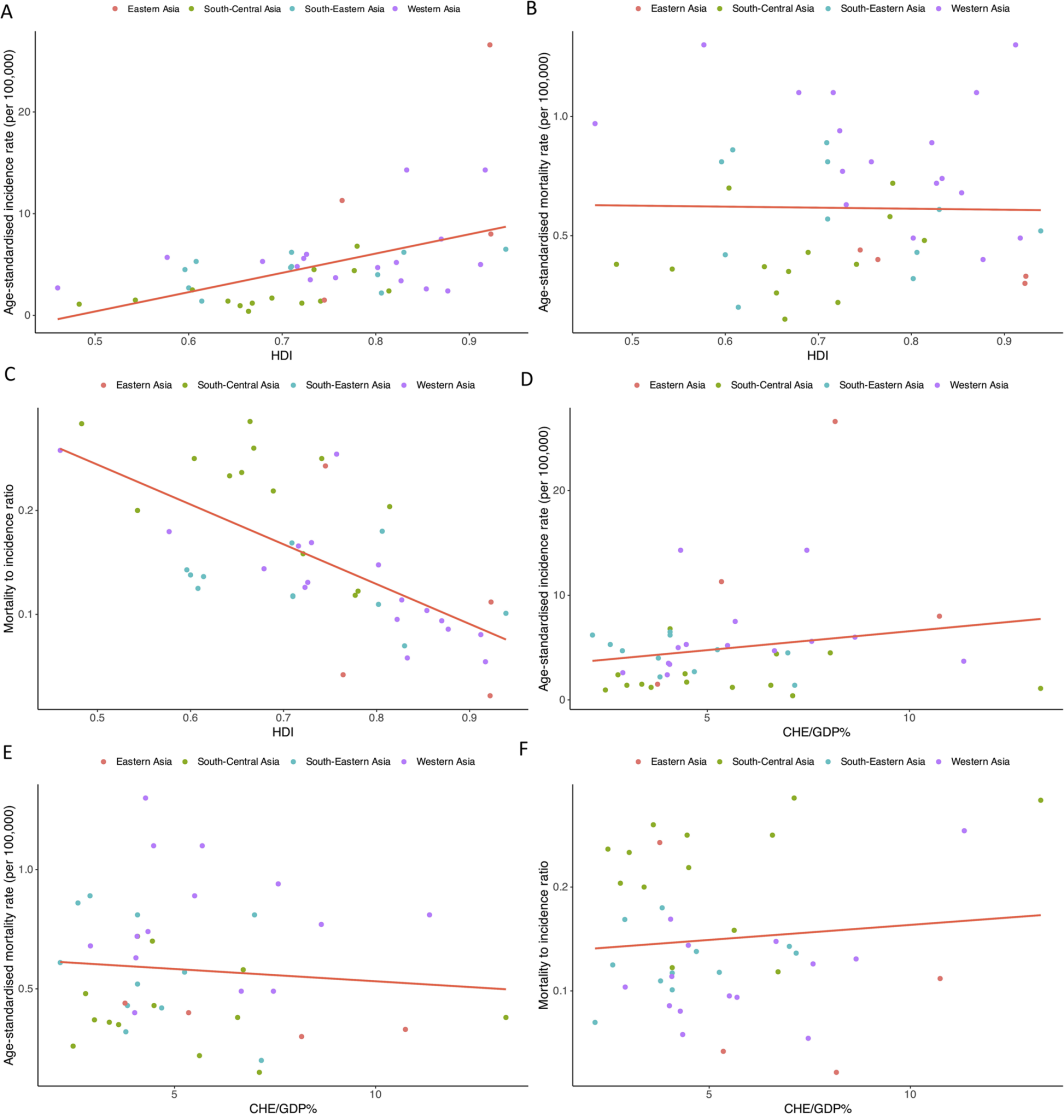
Association of human development index (HDI) with (**A**) age-standardized incidence rate, (**B**) age-standardized mortality rate, and (**C**) mortality-to-incidence ratio. Association of the current healthcare expenditure to gross domestic product (CHE/GDP%) with (**D**) age-standardized incidence rate, **(E)** age-standardized mortality rate, and (**F**) mortality-to-incidence ratio

A moderate, significant positive correlation was observed between thyroid cancer ASIR and the HDI values (Correlation coefficient: 0.490, *p* < 0.001) (Fig. 4A). Also, a significant negative strong correlation was found between HDI and MIR (Correlation coefficient: −0.655; *p* < 0.001) (Fig. 4C). While, there was no significant correlation between HDI and ASMR (Fig. 4B). There were not significant correlations between CHE/GDP% and ASIR (*p* = 0.217) (Fig. 4D), ASMR (*p* = 0.549) (Fig. 4E), and MIR (*p* = 0.516) (Fig. 4F).

### Thyroid cancer trends projection to 2040 in Asia

**Fig. 5:**
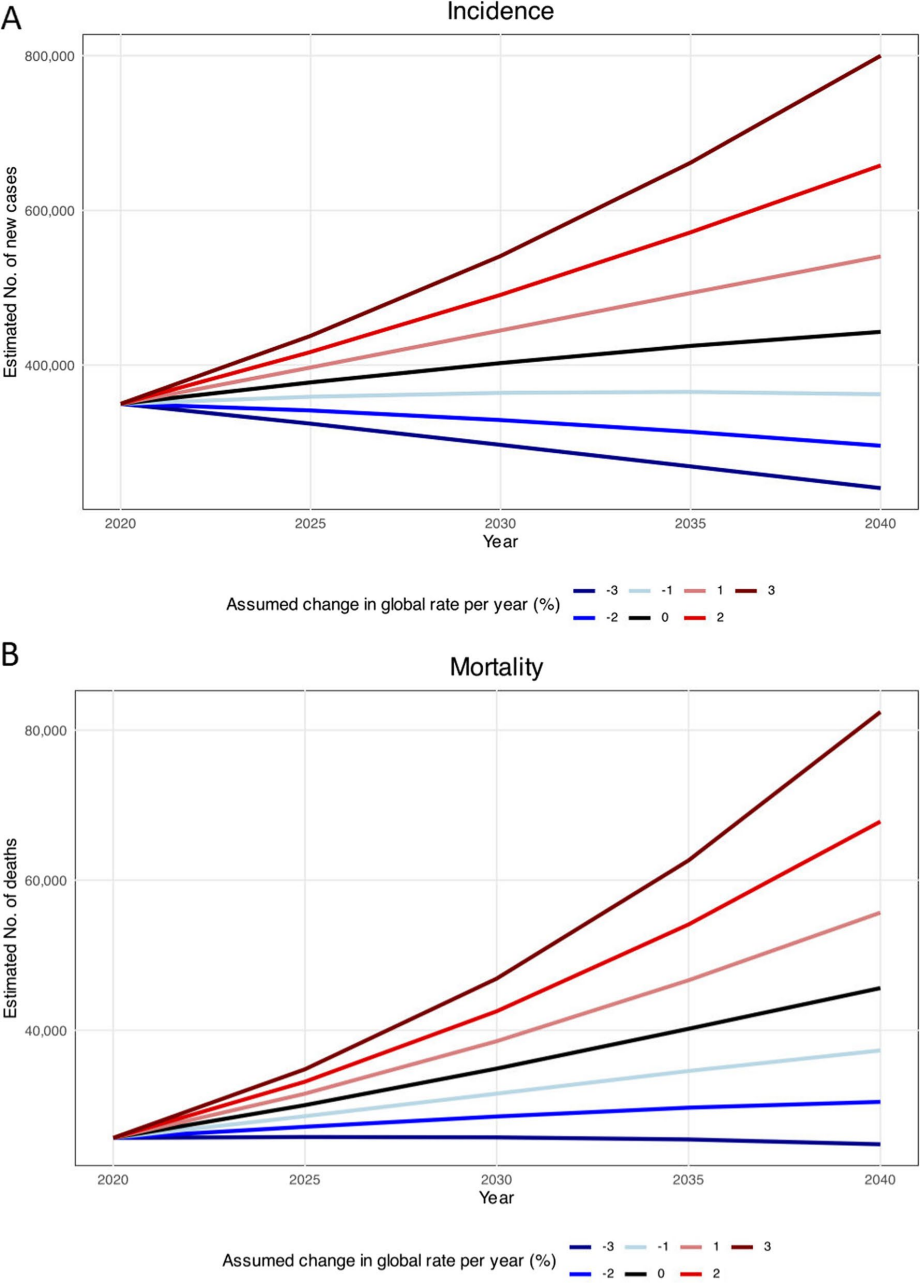
Estimated thyroid cancer (**A**) incidence and (**B**) mortality from 2020 to 2040. The baseline scenario (represented by the black line), posits that there are no alterations in incidence and mortality, meaning that any rise in numbers is solely attributed to changes in population size and composition. Due to the unlikelihood of stable incidence rates, alternative scenarios are provided

The number of newly diagnosed thyroid cancer cases in Asia is expected to rise by 26.6%, going from 349897 cases in 2020 to 443000 cases in 2040 (Fig. 5A). Likewise, thyroid cancer-related mortalities are anticipated to increase by 77.8%, from 25668 in 2020 to 46000 mortalities in 2040, assuming 2020 rates persist (Fig. 5B). In order to see a decrease in thyroid cancer cases in 2040 compared to 2020, reductions in incidence and mortality rates in Asia would need to exceed 1.1% and 2.8%, respectively.

## Discussion

### Main findings

This article presented a recent overview of thyroid cancer epidemiology in Asia using data from the GLOBOCAN 2020 report. Thyroid cancer incidence showed considerable variation across Asia. Elevated incidence rates were observed in several regions, while mortality rates remained low almost everywhere. Incidence rates among females consistently surpassed those of males, although the difference in mortality rates between sexes was much smaller. The study identified the 50–59 year age group as having the highest crude incidence rates of thyroid cancer, whereas the highest mortality rates occurred in those aged 70 and above for both sexes. Significant correlations were also noted between the HDI and both ASIR and the MIR. GLOBOCAN 2020 projected a 26.6% increase in newly diagnosed thyroid cancer cases and a 77.8% rise in mortalities in Asia by 2040.

### Prevalence and Incidence trends, mortality and survival patterns

Over the previous 50 years, the incidence of thyroid cancer has grown considerably worldwide. Despite this, the mortality rates continued to be relatively low [27]. Thyroid cancer ranked as the ninth most often diagnosed cancer globally in 2020, with an estimated 586000 new cases (or 3% of all new cancer cases) [3]. It is responsible for 43646 mortality cases, or 0.4% of all cancer-related deaths globally. By 2040, the numbers of newly diagnosed cases of thyroid cancer and related mortalities in Asia are projected to increase by 26.6% and 77.8%, respectively.

Thyroid cancer is the most frequent endocrine malignancy [28], and it can have an enormous effect on a patient's quality of Life. In 2020, 17 countries in Asia had more than 1000 new cases of thyroid cancer, of which China, India, Japan, and the Republic of Korea were the four countries with the largest number of new patients, accounting for 47.32% of total new thyroid cancer cases worldwide. While incidence rates appeared highest in North America, Eastern Asia, and Oceania, mortality rates were highest in Western Asia, South-Eastern Asia, and Africa. The ASIRs of seven Asian countries (The Republic of Korea, Turkey, Israel, China, Japan, Saudi Arabia, and Sri Lanka) exceeded the global average of 6.6, and the highest ASIRs across Asia were observed in three countries: the Republic of Korea (26.6), Turkey (14.3), and Israel (14.3), all of which were more than twice the global average. However, on a global scale and compared to other continents, Asia had a lower ASIR, following just Africa. There has been a marked surge in incidence rates since the 1980 s in many regions across the world, while mortality rates have remained relatively steady or have shown a decline during the same period [29]. Thyroid cancer has emerged as one of the most rapidly accelerating malignancies. According to a study conducted in the United Kingdom, it ranked as the fastest-accelerating cancer based on average annual percentage change projected from 2015 to 2035, with rates of 2.49% for males and 2.34% for females. These escalations were observed across all age groups [30].

Some studies have suggested that improved accessibility to healthcare facilities, along with advancements in diagnostic technology, may explain the increase in diagnosed cases and incidence rates of thyroid cancer without affecting mortality rates. This aligns with prior research highlighting the association between rising thyroid cancer incidence and the expanding use of advanced diagnostic imaging. These modalities include ultrasonography, the preferred imaging technique for the thyroid gland, along with computed tomography (CT) and magnetic resonance imaging (MRI) [27, 31]. Several factors contribute to this trend. First, the widespread adoption of these imaging tools in recent decades has led to a higher rate of incidental detection of thyroid lesions during investigations for unrelated conditions. Second, the growing accessibility and ease of use of ultrasound have facilitated practices like opportunistic screening and ultrasound-guided fine-needle aspiration biopsy. This trend raises concerns about potential overdiagnosis, as it can identify smaller, asymptomatic nodules that may not progress to clinically significant cancer. To address the risk of overdiagnosis, proactive measures should include the implementation of evidence-based screening guidelines to prevent unnecessary thyroid imaging, particularly in asymptomatic individuals. Additionally, greater efforts should be made to educate both healthcare providers and the public about the implications of overdiagnosis and the potential harms of detecting and treating indolent thyroid lesions. On the other hand, advancements in pathological techniques, increased pathologist scrutiny, and evolving diagnostic criteria have resulted in the detection of a significant number of subclinical, indolent lesions [32]. These may represent a reservoir of slow-growing or non-aggressive cancers. Additionally, healthcare system characteristics can influence detection rates. As highlighted in other studies, privately-oriented healthcare systems and expanding cancer screening programs may lead to the overdiagnosis [29, 33].

Earlier research has shown a significant pool of undetected, small thyroid cancers, particularly the papillary type, found in autopsy studies (ranging from 5 to 36% in both sexes) [34]. This high prevalence suggests that increased scrutiny of the thyroid gland through various techniques can identify these previously unknown tumors within the general population. In the past, most patients were diagnosed with symptoms like noticeable neck masses or nodules causing compression symptoms on the neck. Today, however, a greater percentage of cases are identified through incidental findings of thyroid nodules, often without any noticeable symptoms or a palpable lump. The significant discrepancy between rising incidence rates and stable mortality rates for thyroid cancer strongly suggests that more non-lethal cases are being detected. Estimates suggest that up to one-third of the population may unknowingly harbor such slow-growing or non-aggressive thyroid cancers [27, 35]. The article by Li et al. showed that overdiagnosis accounted for a substantial proportion of thyroid cancer cases globally, with an estimated 75.6% attributable to overdiagnosis, reaching rates above 85% among females in countries like Cyprus, China, South Korea, and Turkey, while being virtually absent in some others such as Uganda, Zimbabwe, and Trinidad and Tobago [36].

The impact of income and sex on the stage of thyroid cancer at which the disease is diagnosed, alongside the positive correlations between thyroid cancer incidence and social and economic indicators such as household income, education, and health insurance, are in line with the hypothesis that the upward trajectory in thyroid cancer incidence is associated with enhanced healthcare accessibility and the adoption of advanced diagnostic methods [33, 37–39].

The precise extent to which the disparity between rising thyroid cancer incidence and stable mortality rates can be attributed to overdiagnosis remains uncertain. Prior studies have attempted to quantify this association. In certain regions, including the Republic of Korea, Belarus, China, Italy, Croatia, Slovakia, and France, estimates suggest that overdiagnosis among women may account for as high as 80% to 95% of newly diagnosed cases between 2008 and 2012. Other areas, such as Denmark, Norway, Ireland, the United Kingdom, and Japan, have reported figures ranging from 50 to 70%. It is noteworthy that a similar trend, albeit to a lesser degree, has been observed in men [40, 41].

Morris et al. [35] reported a notable rise in the incidence of well-differentiated thyroid cancers with palpable size (>2 cm), larger sizes (>4 cm and >6 cm), and those exhibiting extrathyroidal extension or neck metastases. The growing number of cancers with these unfavorable features and across all tumor sizes and histologies strengthens the argument for a genuine rise in thyroid cancer incidence, potentially exceeding what can be solely attributed to overdiagnosis of small, subclinical lesions. To reduce potential risk factors, proactive strategies should focus on reducing environmental exposures to potential carcinogens, promoting dietary interventions to reduce obesity-related risks, and ensuring that individuals are aware of radiation exposure as a modifiable risk factor. Exposure to both recognized and undiscovered hormonal, nutritional, genetic, environmental, and other risk factors could have influenced this observed trend [32]. Prior research has indicated a connection between well-differentiated thyroid carcinoma and environmental contaminants such as hydrocarbons, pesticides, heavy metals, and nitrate [32]. The evolving molecular genetic profile of thyroid cancer, including mutations in genes like *BRAF* and *RAS*, indicates the emergence of new etiologies for this tumor type, possibly of a chemical or dietary nature [42]. By adopting a multi-faceted approach that includes awareness campaigns, targeted screening strategies, and risk reduction initiatives, it may be possible to curb the rising incidence of thyroid cancer while minimizing unnecessary interventions.

Another risk factor to consider is the modern lifestyle. A shift away from traditional diets towards a Western-style diet high in saturated fats and added sugars could be contributing to this trend. Obesity and adiposity, which are often linked to such dietary changes, appear to play a role in comparatively high incidence and mortality rates of thyroid cancer [43]. A study by Kitahara et al. [44] suggests that excess weight significantly contributes to thyroid cancer diagnoses in older adults. Their findings estimate that if overweight and obesity were not risk factors for thyroid cancer, there would have been nearly two and three fewer papillary thyroid carcinoma (PTC) per 100000 individuals aged 60 and over in 2005 and 2015, respectively. The study also claims that one of every six PTCs and nearly two-thirds of large PTCs diagnosed in this age group among the United State adults might be linked to excess weight. It is important to acknowledge, however, that this association could be partially inflated by detection bias. People with obesity-related health problems might undergo more meticulous medical evaluations, potentially leading to earlier detection of thyroid cancer. Given the thyroid gland's sensitivity to radiation, some experts have proposed a link between increased radiation exposure and the observed rise in thyroid cancer. This association might be driven by the extensive use of radiation therapy for benign disorders of the head and neck in children and adolescents, the growing use of diagnostic radiography like CT scans, or exposure to radiation sources like nuclear fallout [33].

To reduce the rising incidence and prevent unnecessary interventions, several measures are recommended: 1) Adopting risk‐stratified screening guidelines that limit the use of cervical ultrasound to individuals with clinical suspicion (e.g., palpable nodules, family history or radiation exposure); 2) Educating healthcare providers on the natural history of papillary thyroid microcarcinoma and on criteria for active surveillance versus immediate intervention; 3) Launching public awareness campaigns to inform asymptomatic individuals about the potential harms of overdiagnosis and overtreatment; and 4) Strengthening registry data quality and integrating tumor‐size reporting to monitor the burden of microcarcinomas over time. These measures might be useful for balancing early detection of clinically significant disease with the reduction of overdiagnosis and its sequelae.

### Regional disparities

The incidence of thyroid cancer exhibits striking variations across Asia, with remarkable differences between and within countries [45]. The ASIRs for thyroid cancer in Asia display a staggering 66.5-fold difference, ranging from the highest rates in the Republic of Korea (26.6 per 100000) to the lowest in Tajikistan (0.4 per 100000). The ASMR shows an 8.67-fold variation across countries with the highest and lowest mortality rates. Despite the absence of major environmental factors like nuclear accidents, South Korea has the highest reported incidence rates. This may be attributed to a healthcare system that emphasizes cancer screening, potentially leading to the overdiagnosis of slow-growing or indolent thyroid cancers [46]. South Korea's healthcare system is extremely dependent on patient out-of-pocket payments and private insurance, creating a less-regulated market susceptible to commercialization. This could lead to overuse of services, including unregulated thyroid cancer screening with ultrasound. In such a privately driven system, both patients seeking excessive examinations and healthcare providers recommending unnecessary tests might contribute to the overdiagnosis of thyroid cancer [33]. While some research suggests a decline in thyroidectomy rates in South Korea since 2014, possibly due to growing awareness of overdiagnosis [40], this country remains at the forefront in terms of both ASIRs and the 5-year prevalence rates of thyroid cancer.

An increasing recognition of the significant implications of overdiagnosis and the slow-growing nature of small thyroid cancers has prompted revisions to international clinical practice guidelines. These revisions discourage routine screening for thyroid cancer and instead advocate for active surveillance of microcarcinoma. The worldwide impact of these guideline changes remains to be fully understood. However, the substantial decrease in thyroid cancer incidence rates observed in South Korea since 2010 and in the United States since 2015 suggests that the growing acceptance of guidelines and an active surveillance strategy may already be alleviating some of the adverse effects associated with overdiagnosis [3].

As previously discussed, the incidence and mortality rates of thyroid cancer exhibit marked disparities across the globe and especially in Asia, with East Asia being a particularly heterogeneous region. Several factors contribute to this phenomenon, including demographic shifts, variations in healthcare systems across Asia, the influence of mass media, and economic disparities between countries [32]. While differences in exposure to established and unknown risk factors (e.g., radiation exposure, obesity, and iodine intake) likely play a role, their contribution is likely modest compared to the other factors mentioned. It is also important to acknowledge that the quality of cancer registries can vary significantly between countries and regions, potentially leading to under- or overestimation of incidence and mortality rates. Interestingly, variation in thyroid cancer incidence exists not only between nations but also within them. In India, for example, despite having some of the world's lowest, a significant tenfold regional disparity exists in both sexes. Regions where there is better accessibility to healthcare seem to have higher and even rising rates of thyroid cancer [47].

### Age and sex patterns

Regarding sex disparities, the incidence rate among females consistently exceeded that of males across various regions, with this disparity being particularly pronounced within the age groups of 40 to 49 and 50 to 59 years old, where the difference can reach up to 17 cases per 100000 people. While rising trends in incidence rates were observed for both sexes, overdiagnosis appears to be more prevalent among women compared to men. Interestingly, the difference between men and women is much smaller in mortality rates [39].

This sex disparity may be partially explained by women's greater demand for healthcare compared to men. Factors such as contraception, pregnancy, and perimenopausal circumstances often lead women to undergo more frequent checkups throughout their lives, providing additional opportunities for the thyroid gland to be examined closely [38, 40, 45, 48]. As a result, it contributes to Asian countries'greater female-to-male ASIR ratio of 3.19.

Our study identified the 50–59 year age group as having the highest crude incidence rates of thyroid cancer across both sexes. This finding aligns with established knowledge that the risk of developing cancer generally increases with advancing age. This age-related rise is likely due to the cumulative effect of risk factors and environmental exposures over time. Furthermore, the trend of global population aging is expected to exacerbate this phenomenon [45]. Advancing age is associated with an increased prevalence of comorbidities, which can complicate the management of thyroid cancer and contribute to higher mortality rates. This is reflected in our findings, which demonstrate the highest thyroid cancer mortality rates in the 70 + age group for both genders.

### Correlations with the socioeconomic status

Evidence suggests significant disparities in both the incidence and mortality rates of thyroid cancer across countries with varying HDI levels. This study investigated the potential role of national development and healthcare expenditure in influencing these disparities, specifically focusing on MIR as an indicator of healthcare and cancer management quality. We hypothesized that more developed regions with higher healthcare spending may have the capacity to deliver more effective thyroid cancer treatment, potentially leading to improved patients’ outcomes and lower MIRs.

The widespread implementation of thyroid cancer screening programs, particularly in developed countries with robust healthcare systems and high healthcare spending, has been Linked to an increased detection of early-stage cancers. As evidenced by data from GLOBOCAN 2020, this trend is exemplified by regions like North America, where a high incidence rate coexists with a lower mortality rate. Conversely, regions like Africa and Western Asia, despite exhibiting the highest ASMRs from thyroid cancer, do not report comparably high ASIRs. In this regard, Northern America has the lowest MIR, and Africa has the highest. These findings suggest that screening programs, while potentially leading to a rise in incidence rates, may also contribute to improved prognoses and lower mortality rates when coupled with appropriate healthcare infrastructure and treatment modalities.

Azadnajafabad and colleagues introduced a novel quality of care index for thyroid cancer to assess cancer care quality across different populations. They found that regions with higher socio-demographic development had better quality of care index scores. Besides that, regions with a high socio-demographic index exhibited the highest incidence rates per 100000 population across all age groups, while low socio-demographic index regions had the lowest incidence rates. Conversely, the mortality rates were highest in low socio-demographic index regions and lowest in high and high-middle socio-demographic index regions. The lower mortality rates in higher socio-demographic index regions were attributed to early detection, better healthcare access, and effective treatment [49]. Lee et al. [33] identified a positive correlation between high GDP per capita and a higher incidence of thyroid cancer. This finding suggests a potential link between national wealth, healthcare financing models, and thyroid cancer detection rates. Supporting this notion, a study by Sung and coauthors reported a significantly higher cancer incidence rate (4.0 times for men and 5.5 times for women) in transitioned countries compared to transitioning countries [3]. Interestingly, these same studies observed minimal variation in mortality rates across different regions in terms of development. Our study's findings echo these observations. We observed significant correlations between the HDI and both ASIR and MIR, suggesting that development level may influence healthcare access and quality. Notably, no significant relationship was found between HDI and ASMR, potentially indicating that advancements in healthcare primarily impact early detection and treatment rather than directly influencing mortality rates from this malignancy.

A recent study by Xu et al. [45] suggests a potential deceleration in the previously observed rapid rise of thyroid cancer incidence rates, particularly in developed countries. This trend likely reflects growing awareness of the potential harms associated with overdiagnosis. Although high-HDI countries exhibit the highest ASIRs, a significant upward trend can be observed in middle- and low-HDI countries. This pattern suggests that overdiagnosis is not solely a concern for developed economies but also warrants attention in resource-limited settings. It is also important to acknowledge that limitations in cancer registration systems in some low- and middle-income countries might lead to delayed and incomplete reporting. This, in turn, could result in an underestimation of both incidence and mortality rates, potentially masking the true burden of thyroid cancer in these regions.

### Challenges and future directions

Our study suggests that the burden of thyroid cancer in Asia is predicted to increase substantially. Annual decreases in incidence and mortality rates exceeding 1.1% and 2.8%, respectively, are necessary to mitigate the future burden of this disease. Considering the projected rise in thyroid cancer incidence, several key areas merit future investigation. These include: 1) refining methods for monitoring disease incidence and analyzing trends; 2) enhancing our understanding of the disease through ongoing research; 3) developing strategies for early and accurate identification of patients amenable to effective treatments; 4) optimizing therapeutic approaches; and 5) implementing targeted prevention plans. Additionally, it is crucial to establish cancer screening protocols that balance the benefits of early detection with the potential harms of overdiagnosis and unnecessary interventions.

Patients continue to undergo aggressive treatments, subjecting them to potential side effects without guaranteed benefits. The detection of small thyroid cancers and the phenomenon of overdiagnosis can lead to healthy individuals being labeled as patients, thereby exposing them to unnecessary therapeutic interventions such as total thyroidectomy, radiotherapy, or neck lymph node dissection [31, 40]. Importantly, many of these thyroid cancers may exhibit an indolent course and pose minimal risk of morbidity or premature mortality. The psychological impact of a cancer diagnosis on both patients and their families is undeniable, and labeling indolent lesions as cancerous can cause significant emotional distress.

Moreover, a significant proportion of patients diagnosed with thyroid cancer, exceeding 90% according to some estimates [46], undergo thyroidectomy. This raises concerns about the potential overtreatment of indolent diseases. Unnecessary surgical procedures can impose a financial burden on healthcare systems and expose patients to potential surgical complications. Furthermore, lifelong thyroid hormone replacement therapy following thyroidectomy necessitates ongoing monitoring and carries its own management considerations. It remains uncertain whether all or most of these patients derive significant benefits from surgical extirpation of what was previously a clinically silent disease. This uncertainty highlights the critical need for more precise diagnostic tools and risk stratification strategies. One of the major challenges in this field is the limited accuracy of cytology in predicting non-classical subtypes of PTC, which has significant difficulties for precise diagnosis and treatment planning. While cytological evaluation is generally reliable for identifying classical PTC, its effectiveness in distinguishing non-classical variants remains suboptimal. This highlights the need for advancements in cytological and imaging techniques to improve the differentiation between aggressive and indolent forms of the disease to guide more appropriate clinical management strategies [50]. Incorporating these advancements into clinical practice could enable us to distinguish patients who are likely to benefit from surgical intervention from those who may be candidates for active surveillance or alternative treatment approaches. By rigorously evaluating the long-term clinical outcomes associated with watchful-waiting strategies compared to surgical intervention through randomized controlled trials and observational cohorts, we can gain valuable insights into the effectiveness of this approach. Findings from such studies could have wide-ranging and significant public health and economic implications. Overdiagnosis of thyroid cancer and subsequent unnecessary treatment lead to not only an additional burden for patients but also wasting limited healthcare expenditures from a societal perspective [33, 40]. Future research endeavors should prioritize providing decision-making frameworks for the ministries of health to enable a thoughtful distribution of medical resources [45].

A strategy to reduce the global and regional disparities in thyroid cancer incidence and mortality rates would be to employ cost-effective interventions tailored to the context of each country. Policymakers should provide particular local solutions to each nation's unique risk factors [45]. We should also consider the long-term socioeconomic advancements in less developed regions of the world. The bulk of existing research in this field draws from data originating in developed countries, potentially limiting its relevance to underdeveloped nations. Hence, it is imperative to undertake population-based cancer registration and novel research in underdeveloped regions [51]. Thorough statistical analysis of the resulting data can subsequently guide the formulation of scientific plans, minimizing the cancer inequities observed between transitioning and transitioned countries.

### Strengths and limitations

Our study's strengths include investigations of epidemiological estimates of thyroid cancer in relation to HDI and CHE/GDP. These findings help identify and map existing disparities in thyroid cancer care, providing valuable insights into the impact of socioeconomic factors and healthcare financing on disease outcomes.

On the other hand, there are several Limitations that should be acknowledged. The accuracy and reliability of national cancer estimates hinge upon the quality of the data sources. According to the GLOBOCAN methodology, the Limitation that requires acknowledgment is the quality, availability, and coverage of cancer data worldwide, especially in low- and middle-income countries. Therefore, careful interpretation of these estimates is crucial. The estimates presented in this study do not consider the impact of the severe acute respiratory syndrome coronavirus 2 (SARS-CoV-2) epidemic. Multiple reports have highlighted disruptions in routine medical appointments, cancer screenings, diagnostic biopsies, surgical procedures, and follow-up care for patients with cancer during the COVID-19 pandemic [52, 53]. Consequently, these disruptions could have affected the registration process for patients with cancer. Furthermore, the evidence supporting the issue of overdiagnosis and overtreatment of low-risk thyroid cancer is primarily based on epidemiological and observational studies. However, these types of evidence are susceptible to confounding factors as well as biases related to selection and reporting, which can impact the reliability and interpretation of the findings. Significant discrepancies in incidence rates exist among different pathological types of thyroid cancer, a factor not captured in the GLOBOCAN database. The absence of histopathology data makes it difficult to understand the exact picture of how different types of thyroid cancer are affecting people around the world. Furthermore, the dataset is missing individual-level information on risk factor exposure, the diagnostic process for each case, and details regarding tumor size or stage at diagnosis. This information would provide additional insights into the extent of overdiagnosis of new cases. Additionally, the accuracy of GLOBOCAN estimates for 2040 could be Limited due to several factors. When utilizing the prediction options, caution is advised. The anticipated number of new cancer cases or deaths in a specific country or region for the years 2025 to 2050 is calculated by multiplying the age-specific incidence or mortality rates from 2020 by the projected populations for those years. Consequently, the aggregate expected numbers of new cancer cases or deaths across various countries or regions for 2025 to 2050 may not align with the overall expected figures if computed as a single entity. Also, these projections rely on incidence and mortality trends from the past years, which may not accurately reflect future trends and can be influenced by an increasing prevalence of risk factors in various regions. The projections did not incorporate changes in the background incidence rate over time, focusing solely on population growth and aging factors. The 2020 GLOBOCAN data was selected for this study as it reflects a baseline unaffected by the significant disruptions caused by the COVID-19 pandemic, such as delays in cancer diagnoses and potential reporting problems. In contrast, the 2022 data may include biases due to changes related to the COVID-19 pandemic, as mentioned by the International Agency for Research on Cancer, which might affect the analysis of underlying cancer trends and associations. Using 2020 data ensures a stable and consistent foundation for evaluating pre-pandemic cancer incidence and mortality patterns. Future studies can consider updating the results based on next iterations of GLOBOCAN data with more accurate primary data after the COVID-19. Moreover, detailed metrics, such as the average annual rate of change in thyroid cancer incidence and mortality were not provided, due to the limited availability of trend data in the GLOBOCAN database for many Asian countries. Future studies should aim to address this gap by using comprehensive trend analyses to better illustrate the changes in the epidemiology of thyroid cancer over time.

## Conclusions

We demonstrated that there are notable sex and age disparities among Asian regions and that incidence rates are directly correlated with countries’ development status. Projections indicate a rising burden by 2040 in Asia. The increasing rates observed in transitioning countries and among younger adults are particularly noteworthy. These findings emphasize the urgent need for proactive measures to mitigate future cases and mortalities associated with thyroid cancer.

## Supplementary Information

Supplementary Material 1

Supplementary Material 2

## Data Availability

The data used for these analyses are available at Global Cancer Observatory, United Nations Development Programme (https://hdr.undp.org/data-center/human-development-index#/indicies/HDI), and Global Health Observatory of World Health Organization [https://www.who.int/data/gho].
