# Supplementary material for: Epidemiology of thyroid cancer in Asia in 2020 and its projection to 2040
Source: BMC Public Health. 2025 Sep 30;25:3201. doi: 10.1186/s12889-025-24029-9 (PMC12486971; doi:10.1186/s12889-025-24029-9)
Supplement: Supplementary file 2 — Supplementary Material 2 [file 12889_2025_24029_MOESM2_ESM.docx]

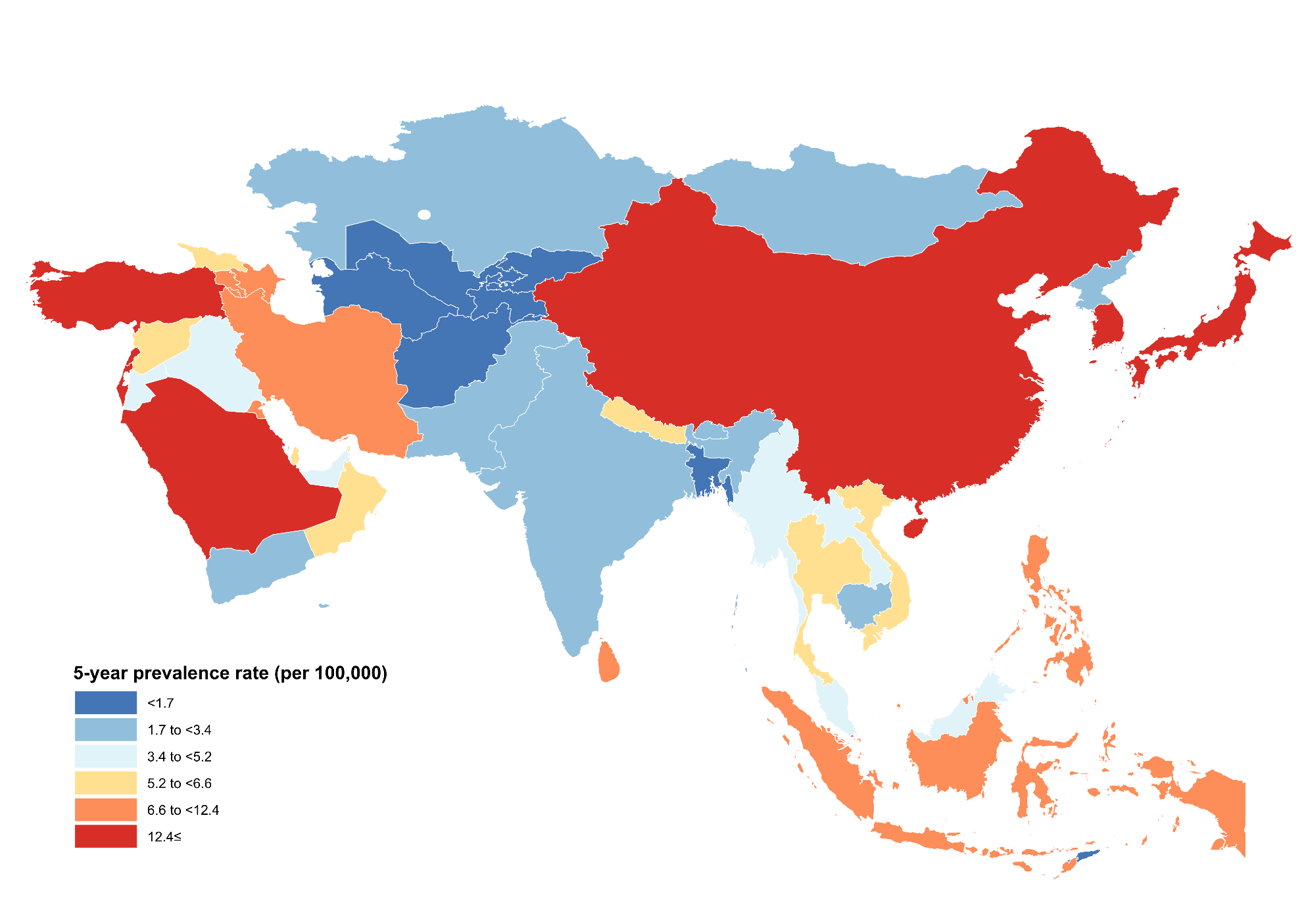


**Figure S1.** Distribution of five-year prevalence rate of male thyroid cancer in 2020 in Asia.


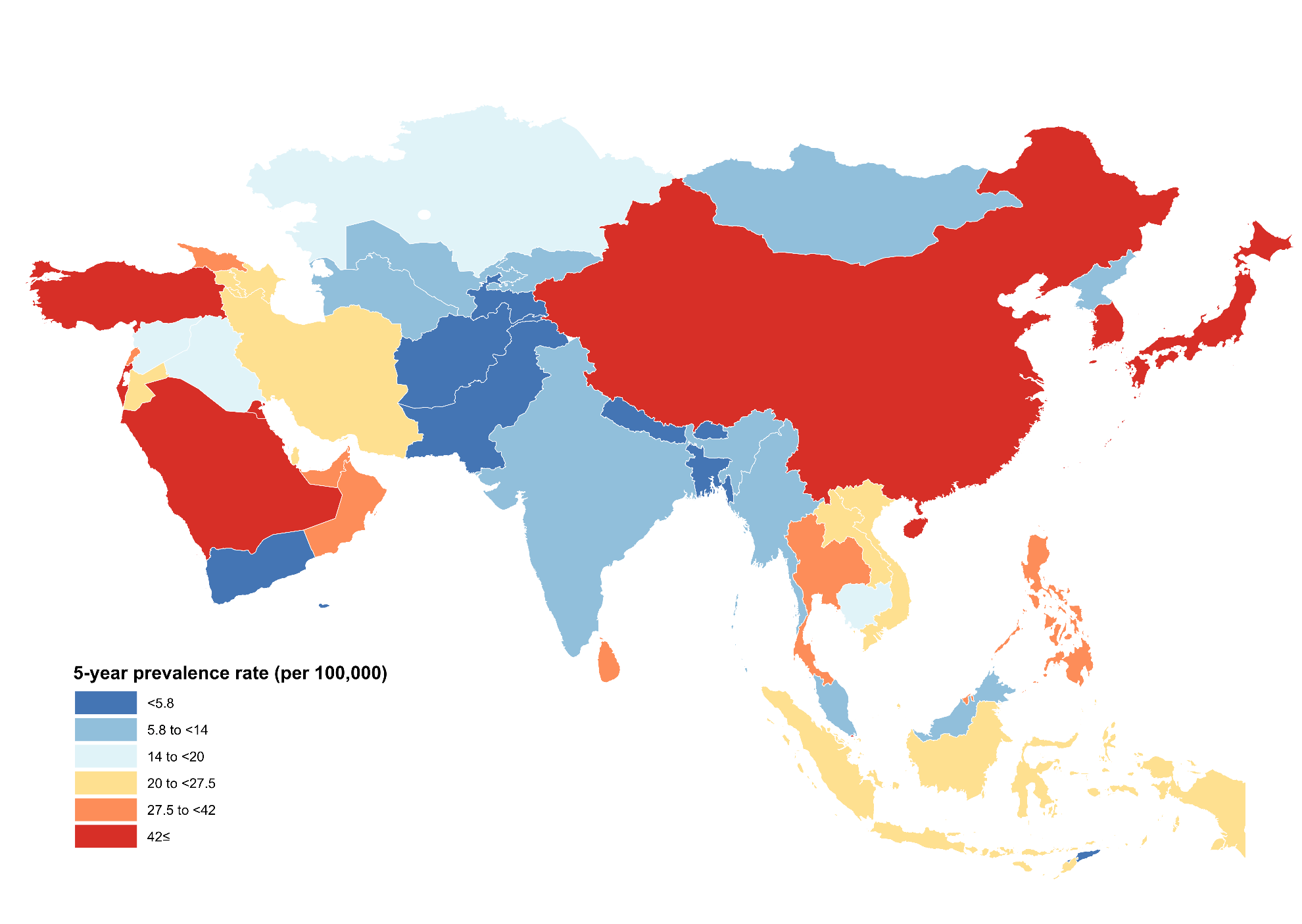


**Figure S2.** Distribution of five-year prevalence rate of female thyroid cancer in 2020 in Asia.


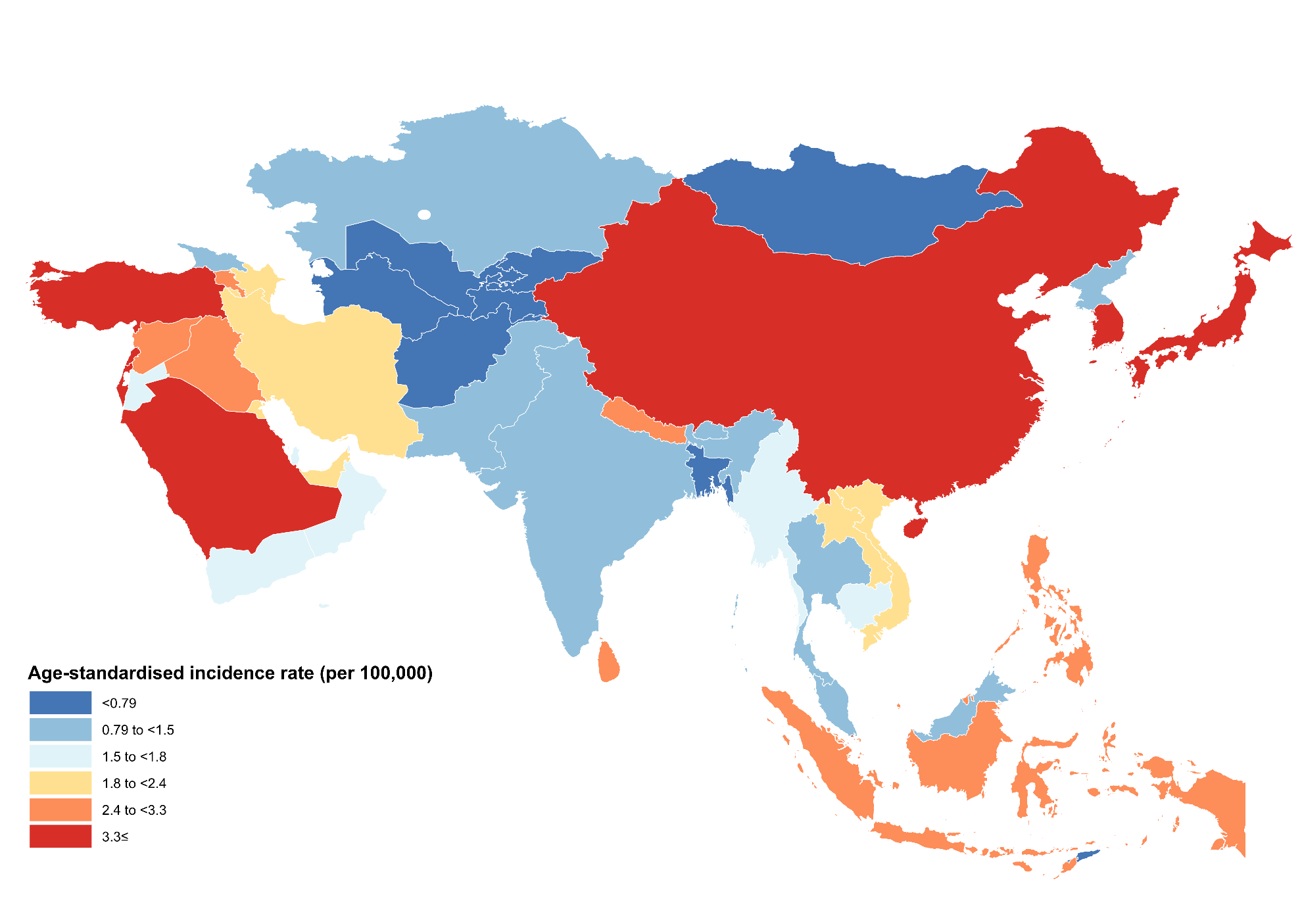


**Figure S3.** Distribution of age-standardized incidence rate of male thyroid cancer in 2020 in Asia.


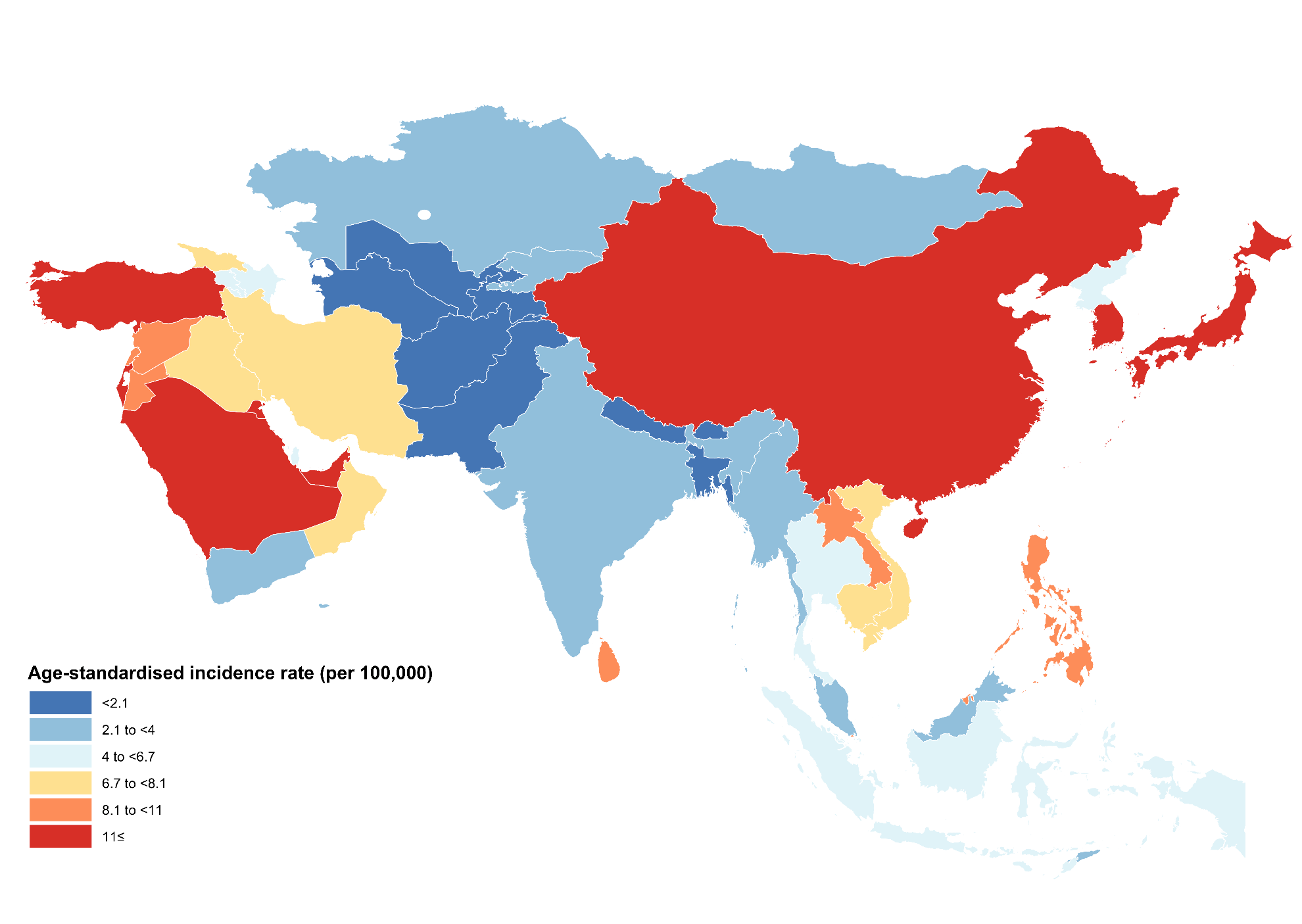


**Figure S4.** Distribution of age-standardized incidence rate of female thyroid cancer in 2020 in Asia.


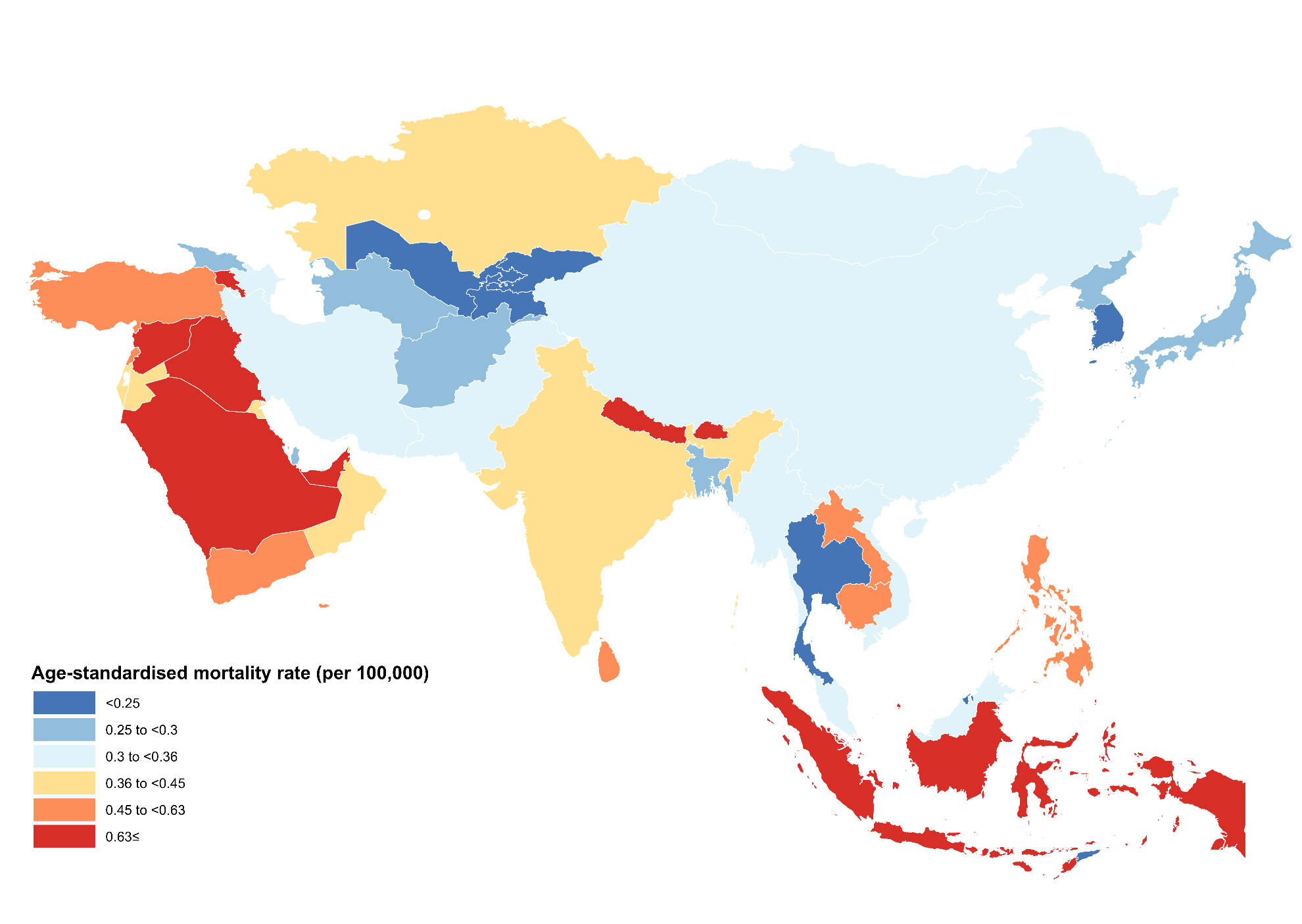


**Figure S5.** Distribution of age-standardized mortality rate of male thyroid cancer in 2020 in Asia.


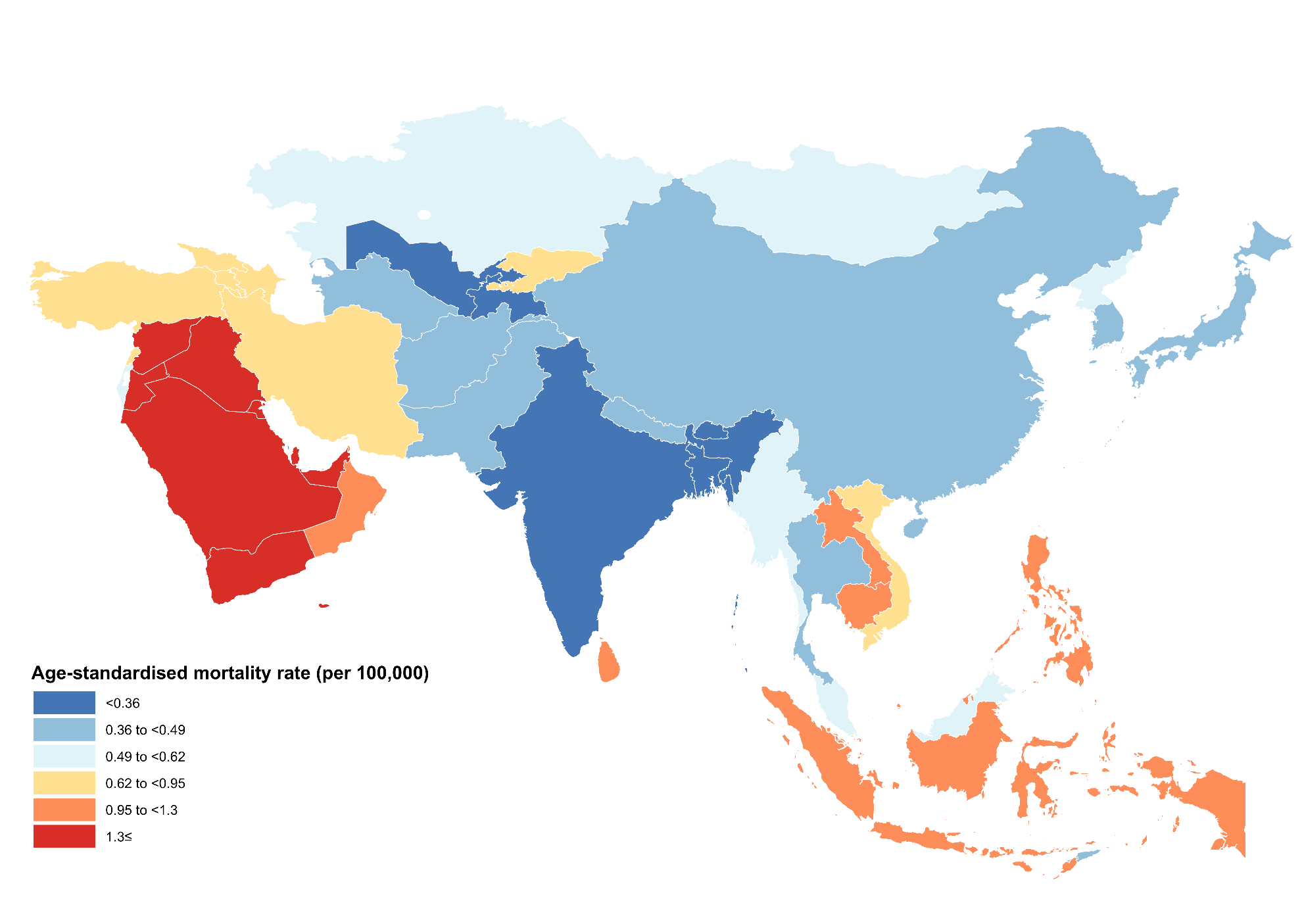


**Figure S6.** Distribution of age-standardized mortality rate of female thyroid cancer in 2020 in Asia.
